# Mendelian Randomization Implicates High-Density Lipoprotein Cholesterol–Associated Mechanisms in Etiology of Age-Related Macular Degeneration

**DOI:** 10.1016/j.ophtha.2017.03.042

**Published:** 2017-08

**Authors:** Stephen Burgess, George Davey Smith

**Affiliations:** 1 MRC Biostatistics Unit, University of Cambridge, Cambridge, United Kingdom; 2 Cardiovascular Epidemiology Unit, University of Cambridge, Cambridge, United Kingdom; 3 MRC Integrative Epidemiology Unit, University of Bristol, Bristol, United Kingdom

**Keywords:** AMD, age-related macular degeneration, CAD, coronary artery disease, CI, confidence interval, HDL, high-density lipoprotein, LDL, low-density lipoprotein, OR, odds ratio

## Abstract

**Purpose:**

Undertake a systematic investigation into associations between genetic predictors of lipid fractions and age-related macular degeneration (AMD) risk.

**Design:**

Two-sample Mendelian randomization investigation using published data.

**Participants:**

A total of 33 526 individuals (16 144 cases, 17 832 controls) predominantly of European ancestry from the International Age-related Macular Degeneration Genomics Consortium.

**Methods:**

We consider 185 variants previously demonstrated to be associated with at least 1 of low-density lipoprotein (LDL) cholesterol, high-density lipoprotein (HDL) cholesterol, or triglycerides at a genome-wide level of significance, and test their associations with AMD. We particularly focus on variants in gene regions that are proxies for specific pharmacologic agents for lipid therapy. We then conduct a 2-sample Mendelian randomization investigation to assess the causal roles of LDL-cholesterol, HDL-cholesterol, and triglycerides on AMD risk. We also conduct parallel investigations for coronary artery disease (CAD) (viewed as a positive control) and Alzheimer's disease (a negative control) for comparison.

**Main Outcome Measures:**

Diagnosis of AMD.

**Results:**

We find evidence that HDL-cholesterol is a causal risk factor for AMD, with an odds ratio (OR) estimate of 1.22 (95% confidence interval [CI], 1.03–1.44) per 1 standard deviation increase in HDL-cholesterol. No causal effect of LDL-cholesterol or triglycerides was found. Variants in the *CETP* gene region associated with increased circulating HDL-cholesterol also associate with increased AMD risk, although variants in the *LIPC* gene region that increase circulating HDL-cholesterol have the opposite direction of association with AMD risk. Parallel analyses suggest that lipids have a greater role for AMD compared with Alzheimer's disease, but a lesser role than for CAD.

**Conclusions:**

Some genetic evidence suggests that HDL-cholesterol is a causal risk factor for AMD risk and that increasing HDL-cholesterol (particularly via *CETP* inhibition) will increase AMD risk.

Age-related macular degeneration (AMD) has one of the longest histories of genetic discovery efforts of any disease in the genome-wide association study era.^1^ To date, genetic variants in 34 independent loci have been demonstrated to be associated with AMD risk,^2^ highlighting several biological mechanisms that provide insight into etiologic processes and may suggest potential therapeutic targets.^3^ Several of the genetic variants associated with AMD risk are located in gene regions that also have associations with lipids or lipid-related biology, in particular the *CETP*, *LIPC,* and *APOE* gene regions. Links between lipid deposition and AMD have been hypothesized for more than 50 years.^4^ High-density lipoprotein (HDL) cholesterol concentrations have been shown to be positively associated with AMD risk in observational studies, whereas low-density lipoprotein (LDL) cholesterol and triglycerides generally have been found to be negatively associated with risk.^5^, ^6^, ^7^ Previous investigators have suggested mechanistic links between atherosclerosis and pathologic features of AMD, such as soft drusen and lipid deposition in Bruch's membrane, using information about the function of lipid-related genetic variants associated with AMD risk.^8^, ^9^ However, links between genetic variants associated with lipid fractions and AMD risk has not been systematically investigated.

Mendelian randomization is the use of genetic variants as proxies for modifiable risk factors.^10^, ^11^ A genetic variant that has a specific association with a risk factor can be used to assess the effect of long-term elevated levels of that risk factor on a disease outcome. The approach exploits the random allocation of genetic variants at meiosis, which results in genetic variants being independently distributed in the population from potential confounders, and the fixed nature of genetic variants, which results in genetic associations being immune to the influences of environmental factors and reverse causation. Mendelian randomization investigations address the causal question: Do long-term elevated levels of the risk factor lead to increased (or decreased) risk of the disease outcome? Previous Mendelian randomization analyses have suggested that LDL-cholesterol is a causal risk factor for coronary artery disease (CAD) risk,^12^, ^13^ but HDL-cholesterol is not.^14^

In this article, we apply a 2-sample Mendelian randomization approach^15^ to consider the effects of lipid fractions on AMD risk using 185 genetic variants previously demonstrated to be associated with at least 1 of LDL-cholesterol, HDL-cholesterol, or triglycerides at a genome-wide level of significance. We consider the associations of these variants with lipid fractions taken from the Global Lipids Genetics Consortium on up to 188 577 individuals of European ancestry^16^ and associations with AMD risk from the International Age-related Macular Degeneration Genomics Consortium on up to 33 526 individuals (16 144 cases, 17 832 controls) predominantly of European ancestry.^2^

The investigation consists of 4 related components. First, we consider whether the 185 variants are more associated with AMD risk than would be expected by chance alone and highlight those variants associated with AMD risk at a Bonferroni-corrected significance threshold. Second, we consider individual genetic variants in gene regions that are proxies for specific pharmacologic agents that have been or are being developed for lipid therapy. Third, we perform univariable Mendelian randomization analyses for the effects of LDL-cholesterol, HDL-cholesterol, and triglycerides on AMD risk, and undertake sensitivity analyses using the MR-Egger^17^ and weighted median^18^ methods that make weaker assumptions than those in a standard Mendelian randomization analysis. Fourth, we perform a multivariable Mendelian randomization analysis for the effects of the lipid fractions on AMD risk.^19^ For part of these analyses, we also test for heterogeneity in the models to see whether genetic associations with AMD risk vary more than would be expected on the basis of the associations of the variants with the lipid fractions alone.

For a positive control, we also consider genetic associations with CAD risk, because lipid fractions are known to influence CAD risk. For a negative control, we consider genetic associations with Alzheimer's disease risk, a disease that has an age profile of cases similar to AMD, but which is not known to be linked to lipid fractions or lipid-related variants^20^ (with the exception of variants in the *APOE* gene region that are strong predictors of Alzheimer's disease^21^). In each analysis, we compare the genetic associations and causal estimates obtained for AMD with those for CAD and Alzheimer's disease. Associations with CAD risk are taken from the Coronary Artery Disease Genome wide Replication and Meta-analysis plus Coronary Artery Disease (CARDIoGRAMplusC4D) consortium on up to 171 191 individuals (60 801 cases, 110 390 controls) mostly of European ancestry.^22^ Associations with Alzheimer's disease risk are taken from the International Genomics of Alzheimer's Project consortium on up to 54 162 individuals (17 008 cases, 37 154 controls) of European ancestry (discovery phase only).^23^

## Methods

All analyses were performed using R (version 3.3.1). All statistical tests are 2 sided. This article used only publically available data and thus did not require specific ethical approval. Ethical approval for the original studies can be found in the original source articles. This research adhered to the Declaration of Helsinki.

### Genetic Associations of Variants with Disease Outcomes

Genetic associations with LDL-cholesterol, HDL-cholesterol, and triglycerides were obtained from the Global Lipids Genetics Consortium^16^; associations with AMD risk were obtained from the International Age-related Macular Degeneration Genomics Consortium^2^; associations with CAD risk were obtained from the CARDIoGRAMplusC4D consortium^22^; and associations with Alzheimer's disease were obtained from the International Genomics of Alzheimer's Project.^23^ Associations with AMD risk are for advanced AMD cases (defined as “geographic atrophy or choroidal neovascularization in at least 1 eye and age at first diagnosis ≥50 years”) versus controls (no advanced or intermediate AMD; intermediate AMD is defined as “pigmentary changes in the retinal pigment epithelium [RPE] or more than 5 macular drusen greater than 63 μm in diameter and age at first diagnosis ≥50 years”).

Beta-coefficients and standard errors for all variants are available for download, except for the associations with AMD risk. For these, we took the *P* values and directions of associations that are published by the International Age-related Macular Degeneration Genomics Consortium (http://csg.sph.umich.edu/abecasis/public/amd2015/), and converted the *P* values to *z* scores. We used published association estimates (beta-coefficients and standard errors) with AMD risk for the 34 genome-wide significant variants (see Table 1 in reference 2), and the assumption that the standard error of the beta-coefficient from a logistic regression analysis is proportional to $1-\sqrt{MAF\left(1-MAF\right)}$, where MAF is the minor allele frequency (assuming that the sample size was the same for all variants).^24^ This means that the standard error multiplied by $\sqrt{MAF\left(1-MAF\right)}$ should be constant for all variants. We took the average value of this expression for the 34 genome-wide significant variants and divided by $\sqrt{MAF\left(1-MAF\right)}$ to estimate the standard errors for the remaining variants. We then multiplied the estimated standard error by the published *z* score to obtain the beta-coefficient for each variant and used the published direction of association to orientate this coefficient.

**Table 1:** Lipid-Related Genetic Variants Associated with Age-Related Macular Degeneration Risk at a Bonferroni-Corrected Level of Significance

Single Nucleotide Polymorphism	Nearest Gene	Association of Variant with… (Beta Coefficient and *P* Value)			
		LDL-c	HDL-c	Triglycerides	AMD Risk
rs1883025 (C)	*ABCA1*	**0.030, *P =* 1×10**^−11^	**0.070, *P =* 6×10**^−66^	**0.022, *P =* 3×10**^−8^	*0.104, P = 2×10*^*−7*^
rs653178 (C)	*ATXN2*	**−0.023, *P =* 2×10**^−9^	**−0.026, *P =* 1×10**^−13^	*0.010, P = 0.004*	*0.064, P = 0.0002*
rs1532085 (G)	*LIPC*	−0.003, *P =* 0.48	**−0.107, *P =* 2×10**^−209^	**−0.031, *P =* 5×10**^−20^	**0.123, *P =* 3×10**^−12^
rs261342 (C)	*LIPC*	0.003, *P =* 0.69	**−0.107, *P =*6 ×10**^−71^	**−0.045, *P =* 4×10**^−14^	**0.117, *P =* 1×10**^−9^
rs9989419 (G)	*CETP*	**−0.028, *P =* 8×10**^−13^	**0.147, *P =* 8×10**^−373^	**−0.024, *P =* 3×10**^−12^	**0.109, *P =* 3×10**^−10^
rs5880 (G)	*CETP*	*−0.047, P = 9×10*^*−7*^	**0.307, *P =* 4×10**^−257^	**−0.048, *P =* 3×10**^−8^	*0.144, P = 6×10*^*−5*^
rs6859 (G)	*PVRL2*	**−0.084, *P =* 1×10**^−101^	*0.018, P =1 ×10*^*−6*^	*−0.014, P = 6×10*^*−5*^	*0.077, P = 8×10*^*−6*^
rs103294 (T)	*LILRB2/LILRA5*	0.007, *P =* 0.12	**0.052, *P =* 4×10**^−33^	−0.002, *P =* 0.61	*0.087, P = 6×10*^*−5*^
rs4465830 (A)	*ZNF335*	−0.009, *P =* 0.06	**0.060, *P =* 4×10**^−42^	**−0.053, *P =* 5×10**^−36^	*0.087, P = 4×10*^*−5*^

Single nucleotide polymorphisms (effect alleles) are those associated with age-related macular degeneration (AMD) risk at a Bonferroni-corrected level of significance (*P* < 0.05/182 = 0.0003). Beta-coefficients for lipid fractions (low-density lipoprotein cholesterol [LDL-c], high-density lipoprotein cholesterol [HDL-c], and triglycerides) represent changes in the lipid fraction per additional copy of the effect allele in standard deviation units. Beta-coefficients for AMD represent log odds ratios (ORs) per additional copy of the effect allele. Boldface indicates association at a genome-wide level of significance (*P* < 5×10^−8^); italics indicate association at a Bonferroni-corrected level of significance. All variants are oriented to the AMD risk-increasing allele.

To assess the validity of this approach, we repeated it first dividing the 34 variants for which beta-coefficients are provided at random into 2 equal groups of 17. We then found the average value of the constant [standard error multiplied by $\sqrt{MAF\left(1-MAF\right)}$] using the first 17 variants and used this to calculate the beta-coefficients for the associations of the remaining 17 variants. We then compared the calculated values of the beta-coefficients for these variants with their values provided by the consortium. The correlation between the calculated and actual values of the beta-coefficients was 0.993. This suggests that the approach was valid and that the beta-coefficients calculated for the 185 lipid-related variants are close to the true values.

### Genetic Proxies for Lipid Therapy

In addition to simple look-ups of the genetic associations for the chosen variants, we also considered the association of genetic risk scores with AMD risk for variants in the *HMGCR* and *NCP1L1* gene regions. These scores were calculated using 3 and 5 variants, respectively, that have been identified to be minimally correlated (*r*^2^ < 0.2) and independently associated with LDL-cholesterol. We performed an inverse-variance weighted Mendelian randomization analysis for the effect of LDL-cholesterol on AMD risk using correlated variants. This is equivalent to testing the association of a genetic risk score with AMD risk, where the score is weighted by the conditional associations of the variants with LDL-cholesterol.^25^ The Mendelian randomization estimates were an odds ratio (OR) of 0.64 (95% confidence interval [CI], 0.42–0.98; *P* = 0.042) per 1 standard deviation increase in LDL-cholesterol for variants in the *HMGCR* gene region and 0.59 (95% CI, 0.32–1.07; *P* = 0.082) for variants in the *NPC1L1* gene region.

### Univariable Mendelian Randomization

Univariable Mendelian randomization analyses are conducted using summarized data on the per allele genetic associations with the risk factor and with the outcome and the Mendelian Randomization package for the R statistical software platform (https://cran.r-project.org/web/packages/MendelianRandomization/). For each of HDL-cholesterol, LDL-cholesterol, and triglycerides, we take all variants associated with the risk factor at a genome-wide level of significance (86 for HDL-cholesterol, 76 for LDL-cholesterol, 51 for triglycerides). The genetic associations with the outcome for those variants are regressed on the genetic associations with the risk factor. Inverse-variance weights are used in the regression model. A (multiplicative) random-effects model is used in each analysis. For the inverse-variance weighted method, the intercept in the regression model is fixed at zero.^15^ For the MR-Egger method, all genetic associations are oriented to the risk factor–increasing allele, and the intercept is estimated as part of the analysis.^17^ In the weighted median method, the same inverse-variance weights are used, and CIs are constructed using a previously described bootstrap method.^18^

### Multivariable Mendelian Randomization

Multivariable Mendelian randomization analyses are conducted using summarized data. A multivariable weighted regression analysis is performed with the genetic associations with the outcome regressed on the genetic associations with the 3 risk factors in a single regression model.^26^ The intercept in this regression model was fixed at zero. Because the variants are allowed to be associated with any or all of the risk factors, data on all of the 185 variants that have associations with the outcome are included in the analysis. Inverse-variance weights are used in the regression model. A (multiplicative) random-effects model is used in each analysis.

## Results

### Genetic Associations of Variants with Disease Outcomes

**Figure 1:**
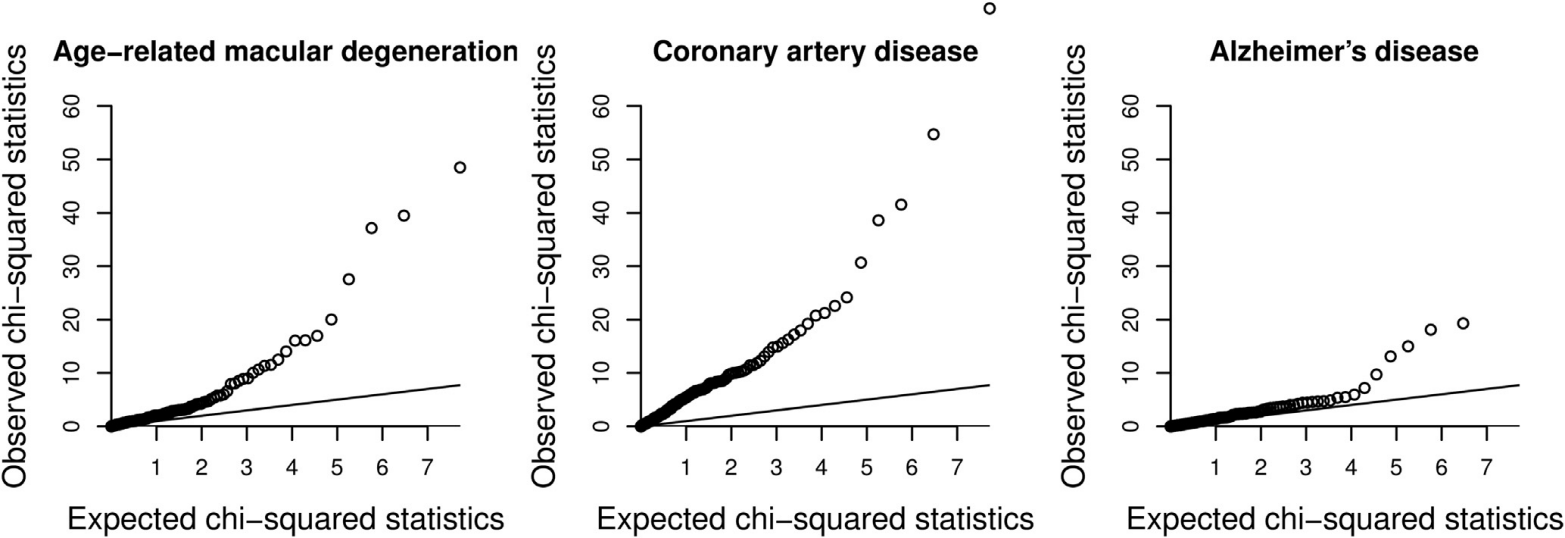
Quantile-quantile plot of chi-square statistics for associations of lipid-related variants with disease outcomes. Chi-square association statistics for 182 genetic variants (3 variants, rs188026950, rs2247056, and rs4332136, were omitted because of missing data) against expected values of a chi-square distribution under the null hypothesis of no association of the variants with disease risk. The line represents the null hypothesis. The *APOE* variant (rs10401969) is omitted from the plot for Alzheimer's disease; its observed chi-square statistic was 435.8.

We constructed a quantile-quantile plot to compare the chi-square statistics for the association with AMD risk with the expected distribution of chi-square statistics under the null for 185 lipid-related variants in 157 different genetic loci that were associated at a genome-wide level of significance with at least 1 lipid fraction in a conditional analysis (3 variants: rs188026950, rs2247056, and rs4332136 were omitted from the analysis because of missing data for at least 1 disease; none were strongly associated with any outcome in the available associations; *P* > 0.05). The plots for AMD, CAD, and Alzheimer's disease are provided in Figure 1. In total, 9 variants were associated with AMD risk at a Bonferroni-corrected *P* value (*P* < 0.05/182 = 0.0003), and 3 variants were associated with AMD risk at a genome-wide level of significance (*P* < 5×10^−8^). This compares with 17 and 5 variants for CAD risk, and 4 and 1 variants for Alzheimer's disease risk. A list of variants associated with AMD risk at a Bonferroni-corrected level of significance is provided in Table 1. Similar lists for CAD and Alzheimer's disease are provided in Tables S1 and S2 (available at www.aaojournal.org).

The lipid-related variants with the strongest associations with AMD risk have a similar strength of statistical association with disease risk as those variants with the strongest associations with CAD risk. The tail of the distribution is longer for CAD, with a greater number of variants associated with CAD risk at sub–genome-wide association study significance levels (e.g., 32 variants were associated with AMD risk at *P* < 0.05, compared with 72 for CAD risk). However, the sample size for genetic associations with CAD risk was larger, meaning that true positive associations with CAD risk are more likely to be detected and make direct comparisons for the total number of lipid-related predictors of AMD and CAD problematic. In contrast, the associations of lipid-related variants with Alzheimer's disease are stronger than would be expected due to chance alone, but not considerably so (19 variants were associated with Alzheimer's disease risk at *P* < 0.05; 9 associations would be expected due to chance alone).

### Genetic Proxies for Lipid Therapy

From this list of 185 variants, we considered variants in the *PCSK9* gene region that can be considered as a proxy for PCSK9 inhibitors,^27^ in the *HMGCR* gene region (proxy for statin treatment^28^), in the *NPC1L1* gene region (proxy for ezetimibe and related NPC1L1 inhibitors^29^), in the *LPA* and *APOC3* gene regions (treatments to lower lipoprotein(a)^30^ and inhibit apolipoprotein C-III^31^ are in development), in the *CETP* gene region (proxy for anacetrapib and related CETP inhibitors^32^), and in the *APOE* gene region (a potential target for pharmacologic intervention). Associations of these variants with AMD risk, CAD risk, and Alzheimer's disease risk are provided in Table 2.

**Table 2:** Genetic Proxies for Lipid Therapy and Their Associations with Disease Outcomes

Single Nucleotide Polymorphism	Gene Region	Association of Variant with… (Beta Coefficient and *P* Value)					
		LDL-c	HDL-c	Triglycerides	AMD Risk	CAD Risk	ALZ Risk
rs12067569 (G)	*PCSK9*∗	**−0.089**	0.007	0.005	0.020	−0.048	−0.048
		***P =* 9×10**^−11^	*P =* 0.33	*P =* 0.52	*P =* 0.61	*P =* 0.04	*P =* 0.25
rs7703051 (C)	*HMGCR*	**−0.073**	−0.002	−0.006	0.044	−0.030	−0.015
		***P =* 5×10**^−85^	*P =* 0.56	*P =* 0.09	*P =* 0.010	*P =* 0.002	*P =* 0.37
rs2297374 (T)	*LPA*	**−0.032**	0.006	−0.009	0.026	*−0.038*	−0.014
		***P =* 6×10**^−18^	*P =* 0.11	*P =* 0.008	*P =* 0.14	*P = 8×10*^*−5*^	*P =* 0.40
rs1564348 (T)	*LPA*	**−0.048**	0.008	−0.016	0.026	−0.030	−0.007
		***P =* 3×10**^−22^	*P =* 0.10	*P =* 0.0003	*P =* 0.28	*P =* 0.022	*P =* 0.74
rs2073547 (A)	*NPC1L1*	**−0.048**	0.005	−0.015	0.021	−0.021	0.025
		***P =* 5×10**^−23^	*P =* 0.28	*P =* 0.0009	*P =* 0.32	*P =* 0.08	*P =* 0.24
rs217386 (A)	*NPC1L1*	**−0.036**	0.001	−0.010	0.031	−0.022	0.005
		***P =* 8×10**^−22^	*P =* 0.71	*P =* 0.003	*P =* 0.08	*P =* 0.029	*P =* 0.77
rs10790162 (A)	*APOC3*	**0.076**	**−0.095**	**0.230**	0.042	0.043	0.046
		***P =* 3×10**^−26^	***P =* 3×10**^−46^	***P =* 1×10**^−276^	*P =* 0.16	*P =* 0.004	*P =* 0.13
rs603446 (C)	*APOC3*	0.009	−0.002	**0.050**	0.006	0.015	0.032
		*P =* 0.013	*P =* 0.60	***P =* 2×10**^−50^	*P =* 0.73	*P =* 0.12	*P =* 0.05
rs9989419 (G)	*CETP*	**−0.028**	**0.147**	**−0.024**	**0.109**	−0.009	0.008
		***P =* 8×10**^−13^	***P =* 8×10**^−373^	***P =* 3×10**^−12^	***P =* 3×10**^−10^	*P =* 0.36	*P =* 0.63
rs5880 (G)	*CETP*	*−0.047*	**0.307**	**−0.048**	*0.144*	−0.007	0.027
		*P = 9×10*^*−7*^	***P =* 4×10**^−257^	***P =* 3×10**^−8^	*P = 6×10*^*−5*^	*P =* 0.75	*P =* 0.52
rs6859 (G)	*APOE*	**−0.084**	*0.018*	*−0.014*	*0.077*	−0.026	**−0.334**
		***P =* 1×10**^−101^	*P = 1×10*^*−6*^	*P = 6×10*^*−5*^	*P = 8×10*^*−6*^	*P =* 0.010	***P =* 9×10**^−97^
rs7254892 (A)	*APOE*	**−0.485**	*0.053*	**0.124**	0.063	−0.078	*−0.250*
		***P =* 8×10**^−365^	*P = 3×10*^*−6*^	***P =* 4×10**^−31^	*P =* 0.19	*P =* 0.009	*P = 1×10*^*−5*^

Single nucleotide polymorphisms (effect alleles) are the lead variants in each gene region taken from the Global Lipids Genetics Consortium. Associations with lipid fractions (low-density lipoprotein cholesterol [LDL-c], high-density lipoprotein cholesterol [HDL-c]. and triglycerides) are in standard deviation units. Associations with disease outcomes (age-related macular degeneration [AMD], coronary artery disease [CAD], Alzheimer's disease [ALZ]) are log odds ratios from logistic regression analyses. Boldface indicates association at a genome-wide level of significance (*P* < 5×10^−8^); italics indicate association at a Bonferroni-corrected level of significance (*P* < 0.05/182 = 0.0003). All variants are orientated to the AMD risk-increasing allele.

∗ The original lead variant reported for the *PCSK9* gene region (rs188026950) was not available in the AMD, CAD, or ALZ consortium data.

Although none of the associations with CAD risk reached statistical significance after correction for multiple testing, variants in all the gene regions apart from the *CETP* region were at least nominally associated with CAD risk (*P* < 0.05). This corresponds to what is known from successful randomized trials of reducing CAD risk with statin treatment,^33^ PCSK9 inhibitors^34^ and ezetimibe,^29^ and unsuccessful trials of *CETP* inhibitors.^35^ In contrast, after accounting for multiple testing, variants in the *APOE* and *CETP* gene regions were associated with AMD risk, suggesting that lowering *APOE* may decrease AMD risk and that inhibiting *CETP* to increase HDL-cholesterol levels may increase AMD risk. In addition, the *HMGCR* variant was associated with AMD risk at a nominal level of significance (*P* = 0.01), suggesting that reducing LDL-cholesterol via statin medication may increase AMD risk. This is in contrast to initial reports suggesting a possible protective effect of statin use for AMD,^36^ although a later larger and more detailed analysis reported different directions of association (both protective and deleterious) for statin use on stratification for baseline lipid concentration and AMD status (exudative vs. nonexudative).^37^ However, individuals who take statins typically would have elevated concentrations of LDL-cholesterol before statin prescription, making interpretation of previous observational studies difficult, and the question of the effect of statins on AMD initiation or progression remains unresolved.^38^ We attempted to refine the findings for variants in the *HMGCR* and *NPC1L1* gene regions using genetic risk scores based on multiple variants in each gene region that have previously been identified^39^; similar results were obtained for the associations of these scores with AMD risk (*P* = 0.042 for *HMGCR*, *P* = 0.082 for *NPC1L1*). We also considered the associations with AMD risk of 2 further variants in the *APOE* gene region^40^: rs7412, the T allele of which tags the *APOE* ε2 variant (per allele OR, 1.59; *P* = 2×10^−42^), and rs429358, the C allele of which tags the APOE ε4 variant (per allele OR, 1.12; *P* = 8×10^−6^). Alzheimer's disease was strongly associated with variants in the *APOE* gene region, but was not associated with variants in any of the other gene regions considered.

### Univariable Mendelian Randomization

We performed univariable Mendelian randomization for each of the lipid fractions in turn. For each analysis, we included all genetic variants that were associated with that lipid fraction at a genome-wide level of significance (*P* < 5×10^−8^). This resulted in up to 76 variants being included in the analysis for LDL-cholesterol, 86 variants for HDL-cholesterol, and 51 variants for triglycerides. In addition to the standard method for Mendelian randomization using summarized data (the inverse-variance weighted method^41^), which assumes that all genetic variants are valid instrumental variables (i.e., not associated with confounders and only associated with the outcome via the lipid fraction under analysis^42^), we also performed the MR-Egger method^17^ (which allows variants to have pleiotropic effects on the outcome not via the risk factor under analysis, provided that such effects are independent of instrument strength), and the weighted median method^18^ (which allows some variants not to be valid instruments, provided that at least 50% of the variants by weight are valid instruments). The inverse-variance weighted method tests for an association between genetic predictors of the exposure and the outcome, whereas the MR-Egger method tests for a dose-response relationship in those associations, and the weighted median method assesses whether the associations with the outcome are evidenced across the majority of variants. Results are presented in Table 3, and the associations are presented graphically in Figure S1 (available at www.aaojournal.org).

**Table 3:** Results from Univariable Mendelian Randomization on Each Lipid Fraction in Turn

Lipid Fraction	Method	AMD	CAD	ALZ∗
LDL-c	Inverse-variance weighted	0.94 (0.82–1.09)	1.53 (1.40–1.67)	1.02 (0.93–1.12)
		*P =* 0.41	*P =* 2×10^−14^	*P =* 0.67
	MR-Egger	0.88 (0.70–1.11)	1.61 (1.39–1.85)	1.05 (0.89–1.24)
		*P =* 0.28	*P =* 9×10^−9^	*P =* 0.56
	Weighted median	0.91 (0.80–1.02)	1.59 (1.45–1.73)	1.04 (0.92–1.18)
		*P =* 0.11	*P =* 3×10^−25^	*P =* 0.49
HDL-c	Inverse-variance weighted	1.22 (1.03–1.44)	0.85 (0.76–0.95)	0.98 (0.88–1.09)
		*P =* 0.02	*P =* 0.005	*P =* 0.76
	MR-Egger	1.08 (0.82–1.42)	1.10 (0.93–1.31)	0.91 (0.76–1.09)
		*P =* 0.58	*P =* 0.26	*P =* 0.32
	Weighted median	1.46 (1.23–1.74)	0.95 (0.87–1.05)	1.05 (0.92–1.20)
		*P =* 3×10^−5^	*P =* 0.32	*P =* 0.46
Triglycerides	Inverse-variance weighted	0.85 (0.67–1.07)	1.30 (1.17–1.45)	0.98 (0.88–1.09)
		*P =* 0.16	*P =* 1×10^−5^	*P =* 0.68
	MR-Egger	1.31 (0.93–1.86)	1.09 (0.93–1.29)	1.06 (0.89–1.27)
		*P =* 0.13	*P =* 0.31	*P =* 0.51
	Weighted median	1.13 (0.95–1.34)	1.25 (1.12–1.38)	1.00 (0.85–1.17)
		*P =* 0.17	*P =* 3×10^−5^	*P =* 0.97

ALZ = Alzheimer's disease; AMD = age-related macular degeneration; CAD = coronary artery disease; HDL-c = high-density lipoprotein cholesterol; LDL-c = low-density lipoprotein cholesterol; MR = Mendelian randomization.

Estimates are odds ratios (95% confidence intervals) for the effect of a 1 standard deviation increase in the lipid fraction. All genetic variants that are associated with the lipid fraction at a genome-wide level of significance (*P* < 5×10^−8^) are included in the analysis for that lipid fraction.

∗ Variants from the *APOE* gene region were omitted from analyses for Alzheimer's disease because they dominated the results.

**Figure 2:**
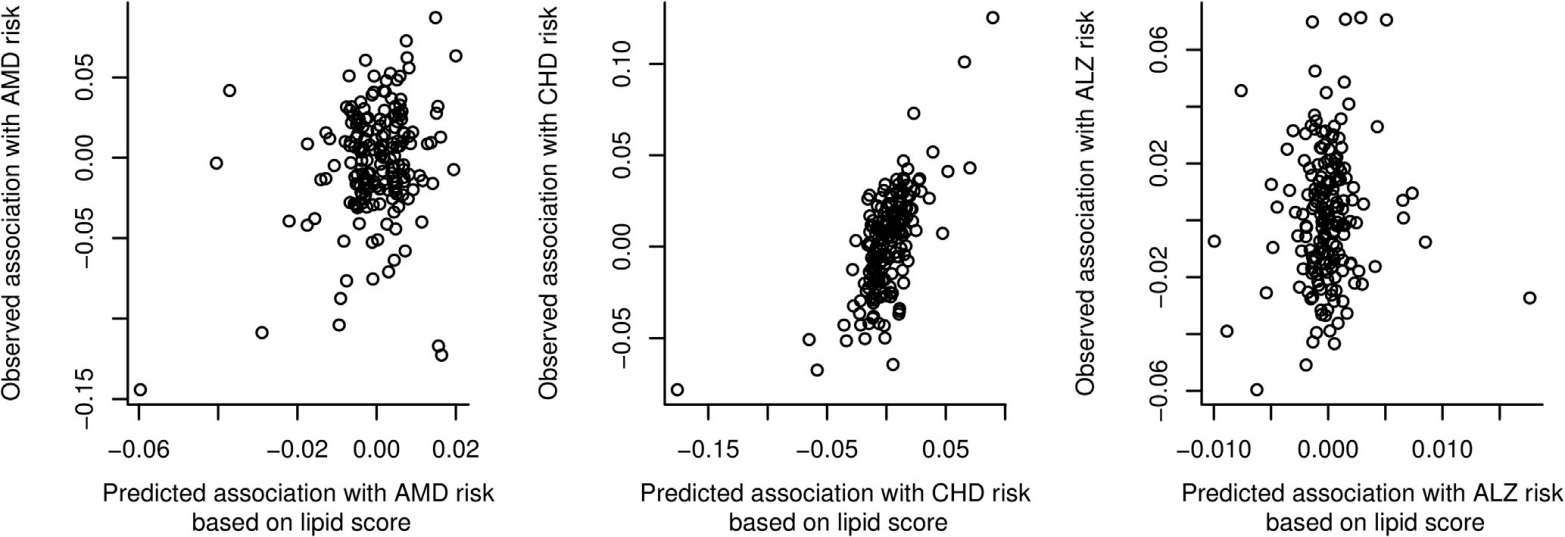
Observed versus expected genetic associations with outcome from multivariable Mendelian randomization analysis. Associations with the outcome for each genetic variant (y-axis) are plotted against the expected associations with the outcome based on the associations with the lipid fractions (fitted values from the multivariable Mendelian randomization regression model; x-axis). Each point represents a single genetic variant. ALZ = Alzheimer's disease; AMD = age-related macular degeneration; CHD = coronary heart disease.

By using the standard inverse-variance weighted method, all 3 lipid fractions are causally associated with CAD risk. The association for LDL-cholesterol is evidenced by all 3 univariable methods, whereas the association for HDL-cholesterol is null in the MR-Egger and weighted median methods, suggesting that the association in the inverse-variance weighted method may be due to pleiotropy. In contrast, although there is little evidence from univariable Mendelian randomization analyses that LDL-cholesterol or triglycerides influence AMD risk, the inverse-variance weighted and weighted median methods suggest a causal effect of HDL-cholesterol on AMD risk, with estimates implying a detrimental effect of increasing HDL-cholesterol on AMD risk. The MR-Egger estimate was somewhat attenuated and compatible with the null, although there was no clear evidence for directional pleiotropy in the MR-Egger intercept test (*P* = 0.28). There was evidence for substantial heterogeneity in the single nucleotide polymorphism–specific causal estimates for HDL-cholesterol, with Cochran's heterogeneity test statistic taking a value of Q = 330.3 (85 degrees of freedom; *P* < 0.0001).^43^ Visual inspection of Figure 2 demonstrates this heterogeneity, with most HDL-cholesterol–increasing genetic variants being associated with increased AMD risk, but some variants showing the opposite direction of association. The 2 variants that are the clearest outliers in their associations with HDL-cholesterol and AMD risk are rs1532085 and rs261342, both located in the *LIPC* gene region (Table 1), which are associated with decreased HDL-cholesterol concentrations and increased AMD risk. Cook's distance (a measure of influence in a regression analysis) was less than 0.15 for all variants except for those in the *CETP* and *LIPC* gene regions. The inverse-variance weighted method for HDL-cholesterol omitting variants in the *CETP* and *LIPC* gene regions from the analysis gave a causal estimate of 1.34 (95% CI, 1.14–1.58), suggesting that the positive Mendelian randomization finding is not driven solely by a small number of variants. In a sensitivity analysis, we performed 1 million iterations in which we removed 30% of genetic variants (28/86) at random from the Mendelian randomization analysis for HDL-cholesterol and re-ran the analysis using the remaining 60 variants; 96% of the iterations reported a positive causal estimate (Fig S2, available at www.aaojournal.org). For Alzheimer's disease, although the univariable Mendelian randomization analyses including all variants suggested a causal effect of LDL-cholesterol on Alzheimer's disease risk, all associations attenuated to the null when the *APOE* variants were omitted from the analysis.

### Multivariable Mendelian Randomization

We performed multivariable Mendelian randomization for all 3 lipid fractions in a single analysis model (Table 4). Multivariable Mendelian randomization allows genetic variants to have pleiotropic effects, but only on other variables included in the model (so a variant is allowed to influence multiple lipid fractions, so long as any association with the outcome is via a lipid fraction).^19^ The analyses each include all of the 185 variants for which data are available on associations with the outcome. The results obtained were similar to those in the univariable analyses, except that only LDL-cholesterol and triglycerides (and not HDL-cholesterol) were associated with CAD risk. Only HDL-cholesterol was associated with AMD risk. None of the 3 lipid fractions were associated with Alzheimer's risk (once variants in the *APOE* gene region were omitted from the analysis).

**Table 4:** Results from Multivariable Mendelian Randomization on All Lipid Fractions in Single Model

Lipid Fraction	Method	AMD	CAD	ALZ∗
LDL-c	Multivariable MR	0.96 (0.83–1.11)	1.48 (1.36–1.61)	1.02 (0.93–1.14)
		*P =* 0.55	*P =* 2×10^−21^	*P =* 0.58
HDL-c	Multivariable MR	1.18 (1.01–1.38)	0.93 (0.85–1.02)	0.94 (0.84–1.05)
		*P =* 0.03	*P =* 0.13	*P =* 0.25
Triglycerides	Multivariable MR	1.04 (0.77–1.12)	1.16 (1.04–1.29)	0.93 (0.82–1.07)
		*P =* 0.44	*P =* 0.009	*P =* 0.31

ALZ = Alzheimer's disease; AMD = age-related macular degeneration; CAD = coronary artery disease; HDL-c = high-density lipoprotein cholesterol; LDL-c = low-density lipoprotein cholesterol; MR = Mendelian randomization.

Estimates are odds ratios (95% confidence intervals) for the effect of a 1 standard deviation increase in the lipid fraction. All genetic variants that are associated with at least 1 lipid fraction at a genome-wide level of significance (*P* < 5×10^−8^) are included in the analyses.

∗ Variants from the *APOE* gene region were omitted from analyses for Alzheimer's disease because they dominated the results.

We plotted fitted values from the multivariable Mendelian randomization analysis models against their associations with the outcome in Figure 2. The fitted values represent the expected associations of the genetic variants with the outcome based on their associations with the lipid fractions alone. In the absence of heterogeneity and pleiotropy, and with infinitely large sample sizes, these graphs would be straight lines through the origin. For CAD risk, correlation between the observed and expected associations with the outcome in Figure 2 is apparent, and the multivariable Mendelian randomization model explains a large proportion of the variance in the observed associations with the outcome (*R*^2^ = 42.4%). In contrast, for AMD risk, the model explains 5.2% of the variance, and for Alzheimer's disease risk, 1.0%, no more than would be expected by chance alone. Genetic predictors of lipid fractions are clearly individually associated with AMD risk, but overall the associations with LDL-cholesterol, HDL-cholesterol, and triglycerides are far less predictive of AMD risk than they are for CAD risk.

## Discussion

In this article, we have analyzed a comprehensive set of variants that have been demonstrated to be associated with at least 1 lipid fraction among LDL-cholesterol, HDL-cholesterol, or triglycerides at a genome-wide level of significance. We have examined the associations of these variants with AMD risk, both considering individual variants that are proxies for lipid therapy or strongly associated with AMD risk, and considering all variants associated with a particular lipid fraction and their association with AMD risk in a Mendelian randomization framework. Two-sample Mendelian randomization was performed using summary statistics on genetic associations with the risk factor and with the disease outcome taken from separate datasets. The standard inverse-variance–weighted analysis is equivalent to considering whether a genetic risk score, weighted by its associations with the risk factor estimated in the first data set, is associated with the outcome in the second data set.^25^ We also considered methods that make alternative assumptions to the standard Mendelian randomization method, including the MR-Egger, weighted median, and multivariable Mendelian randomization methods.

Finally, in addition to AMD, we also considered CAD and Alzheimer's disease as outcomes. Coronary artery disease represents a positive control, which is expected to be associated with lipid-related variants, whereas Alzheimer's disease represents a negative control, because no association with lipid-related variants (aside from variants in the *APOE* gene region) has been previously demonstrated. Although the 185 lipid-related variants were more associated with Alzheimer's disease than would be expected by chance alone, calling into question whether Alzheimer's disease is truly a negative control for lipid fractions, it is perhaps unreasonable to assume that so many variants associated with lipid concentrations should all have null associations with Alzheimer's disease, particularly because several of the variants are associated with other potential risk factors for Alzheimer's disease. However, there was no robust association of any lipid fraction with Alzheimer's disease in any of the Mendelian randomization analyses, suggesting that there is no consistent relationship between lipid-related variants and Alzheimer's disease, and that associations of individual variants with Alzheimer's disease are unlikely to be driven by lipids.

We observed that variants in gene regions linked with HDL-cholesterol–related mechanisms were associated with AMD risk. This was evident through associations with variants in the *CETP* gene region, which can be considered as proxies for *CETP* inhibition. *CETP* inhibition increases HDL-cholesterol levels, and the *CETP* variants associated with increases in HDL-cholesterol concentrations also were associated with increased AMD risk. In addition, variants in the *APOE* gene region that also increase HDL-cholesterol were also associated with increased AMD risk. Although variants in the *CETP* and *APOE* gene regions are also associated with LDL-cholesterol levels, associations with AMD risk were stronger for variants in the CETP gene region, in line with the associations of the variants with HDL-cholesterol (stronger for the *CETP* variants) and the opposite of the associations of the variants with LDL-cholesterol (stronger for the *APOE* variants). Associations of HDL-cholesterol with AMD risk were observed in the Mendelian randomization analyses, specifically using the inverse-variance weighted, weighted median, and multivariable Mendelian randomization methods. Although the association in the MR-Egger method was somewhat attenuated and compatible with the null, there was no clear evidence of directional pleiotropy in the MR-Egger analysis. However, there was substantial heterogeneity in these analyses, suggesting that not all mechanisms for increasing HDL-cholesterol would be expected to increase AMD risk uniformly. For example, the causal estimate using the *CETP* variant rs5880 is an OR of 2.10 (95% CI, 1.30–3.39) per standard deviation increase in HDL-cholesterol, larger than the causal estimate of 1.22 (95% CI, 1.03–1.44) from the Mendelian randomization analysis using all HDL-cholesterol–associated variants. Variants in the *LIPC* gene region that were strongly associated with AMD risk had a direction of association with HDL-cholesterol opposite that of other variants. Overall, the level of evidence from Mendelian randomization for a causal role of long-term elevated levels of HDL-cholesterol in increasing AMD risk is not as strong as that for LDL-cholesterol in CAD, but there is broadly consistent evidence for a causal role across a range of analysis methods.

### Study Strengths and Limitations

An advantage of the Mendelian randomization framework using multiple genetic variants is the ability to consider multiple independent genetic variants in different locations on the genome that each influence HDL-cholesterol and their associations with AMD risk. A disadvantage of the use of multiple variants is that the analysis does not point to a single mechanism as causal for AMD risk, although the *CETP* gene region suggests 1 potential mechanism. A search of the U.S.-based clinicaltrials.gov and the EU Clinical Trials Register did not reveal any trials of *CETP* inhibition that measured AMD as an outcome. The assumption of Mendelian randomization that all genetic variants are valid instrumental variables is unlikely to hold in this case. We were able to consider 3 separate methods that allow for weaker assumptions: the MR-Egger method that allows for unmeasured pleiotropy; the weighted median method, which is robust to the influence of outliers; and the multivariable Mendelian randomization method that allows for measured pleiotropy. These methods generally supported a causal effect of HDL-cholesterol on AMD risk. In addition, the negative control analysis of Alzheimer's disease did not suggest that any of the lipid fractions were causal for Alzheimer's disease, meaning that pleiotropy did not lead to false-positive findings in this case.

The risk factor considered in this investigation was circulating levels of serum lipid concentrations. It seems that AMD-related lipid deposition is influenced both by circulating levels (that underlie systemic processes such as atherosclerosis) and by retina-specific processes.^44^ Intracellular lipid concentrations or lipid concentrations localized to the retina would be more relevant measurements for understanding AMD risk, and heterogeneity in the genetic associations (even different directions of association with AMD risk) may result from discordancy between genetic associations with circulating and intracellular lipid concentrations. Many of the lipid-related genetic variants we use, identified from studies of circulating lipids, have been investigated as specific risk indicators in AMD.^45^ Molecular genetic variation around particular genes could generate effects on retinal lipid processing that are not reflected in, or indeed could be the reverse of, effects on circulating lipids. Speculatively, such mechanisms could explain the opposite associations of *CETP* and *LIPC* variants with AMD in relation to their association with circulating HDL-cholesterol. The local retinal functional consequences of the molecular genetic variation we examine in this study are not well defined and certainly constitute an important area for future research.^44^, ^45^

This analysis used state-of-the-art methods for Mendelian randomization and the largest currently available sources of data on genetic associations with lipid fractions and with AMD risk to address the question of causality as comprehensively as possible, but clearly will benefit from increased functional understanding of genetic variants from which we leverage explanatory power.

In conclusion, some genetic evidence suggests that HDL-cholesterol is a causal risk factor for AMD risk and that increasing HDL-cholesterol (in particular via inhibition of *CETP* and lowering of *APOE*) will lead to increased risk of AMD. Studies developing pharmacologic interventions for lipid therapy should monitor AMD events as a potential adverse outcome, particularly for drugs designed to increase HDL-cholesterol levels and for studies of individuals at a high risk of developing AMD.
