# Supplementary material for: Mendelian Randomization Implicates High-Density Lipoprotein Cholesterol–Associated Mechanisms in Etiology of Age-Related Macular Degeneration
Source: Ophthalmology. 2017 Aug;124(8):1165–74. doi: 10.1016/j.ophtha.2017.03.042 (PMC5526457; doi:10.1016/j.ophtha.2017.03.042)

Supplementary Table 1: Lipid-related genetic variants associated with CAD risk at a Bonferroni-corrected level of significance

| SNP | Nearest gene | Association of variant with… [beta coefficient (standard error)] | | | |
|---|---|---|---|---|---|
| | | LDL-c | HDL-c | Triglycerides | CAD risk |
| rs646776 | *CELSR2* | **0.160 (0.004)** | **-0.034 (0.004)** | 0.003 (0.004) | **0.101 (0.011)** |
| rs1367117 | *APOB* | **0.119 (0.004)** | **-0.022 (0.004)** | **0.025 (0.004)** | *0.041 (0.011)* |
| rs515135 | *APOB* | **0.139 (0.005)** | -0.011 (0.004) | *0.019 (0.004)* | **0.068 (0.012)** |
| rs6544713 | *ABCG8* | **0.081 (0.004)** | -0.003 (0.004) | 0.013 (0.004) | *0.051 (0.010)* |
| rs4148218 | *ABCG8* | **0.044 (0.005)** | -0.003 (0.004) | 0.004 (0.004) | *0.050 (0.012)* |
| rs4530754 | *CSNK1G3* | **-0.028 (0.004)** | -0.001 (0.003) | -0.002 (0.003) | *0.035 (0.009)* |
| rs998584 | *VEGFA* | 0.000 (0.004) | **-0.026 (0.004)** | **0.029 (0.004)** | *0.042 (0.010)* |
| rs2297374 | *SLC22A1* | **0.032 (0.004)** | -0.006 (0.004) | 0.009 (0.003) | *0.038 (0.010)* |
| rs2954022 | *TRIB1* | **0.055 (0.004)** | **-0.040 (0.003)** | **0.078 (0.003)** | *0.043 (0.009)* |
| rs579459 | *ABO* | **0.066 (0.004)** | 0.014 (0.004) | -0.014 (0.004) | **0.073 (0.011)** |
| rs2068888 | *CYP26A1* | *0.017 (0.004)* | **-0.019 (0.004)** | **0.024 (0.003)** | *0.039 (0.010)* |
| rs12801636 | *PCNX3/ PCNXL3* | -0.008 (0.004) | **-0.024 (0.004)** | *0.018 (0.004)* | *0.050 (0.011)* |
| rs653178 | *ATXN2* | **-0.023 (0.004)** | **-0.026 (0.004)** | 0.010 (0.003) | **0.064 (0.010)** |
| rs1169288 | *HNF1A* | **0.038 (0.004)** | 0.010 (0.004) | 0.002 (0.004) | *0.047 (0.010)* |
| rs952044 | *Intergenic* | -0.003 (0.004) | **-0.023 (0.004)** | 0.010 (0.004) | *0.043 (0.010)* |
| rs6511720 | *LDLR* | **0.221 (0.006)** | *-0.025 (0.006)* | 0.008 (0.006) | **0.125 (0.017)** |
| rs688 | *LDLR* | **0.054 (0.004)** | -0.011 (0.003) | 0.004 (0.003) | *0.037 (0.010)* |

SNPs are those associated with coronary artery disease (CAD) risk at a Bonferroni-corrected level of significance ($p < 0.05/182 = 0.0003$). Associations with lipid fractions (low-density lipoprotein cholesterol [LDL-c], high-density lipoprotein cholesterol [HDL-c] and triglycerides) are in standard deviation units. Associations with age-related macular degeneration are log odds ratios from logistic regression analyses. Bold face indicates association at a genome-wide level of significance ($p < 5 \times 10^{-8}$); italics indicates association at a Bonferroni-corrected level of significance. All variants are orientated to the CAD risk-increasing allele.

Supplementary Table 2: Lipid-related genetic variants associated with ALZ risk at a Bonferroni-corrected level of significance

| SNP | Nearest gene | Association of variant with… [beta coefficient (standard error)] | | | |
|---|---|---|---|---|---|
| | | LDL-c | HDL-c | Triglycerides | ALZ risk |
| rs1883025 | *ABCA1* | **-0.030 (0.004)** | **-0.070 (0.004)** | **-0.022 (0.004)** | *0.070 (0.018)* |
| rs17788930 | *FNBP4* | 0.005 (0.004) | **0.036 (0.004)** | -0.011 (0.004) | *0.070 (0.016)* |
| rs6859 | *APOC* | **0.084 (0.004)** | **-0.018 (0.004)** | *0.014 (0.004)* | **0.334 (0.016)** |
| rs7254892 | *APOC* | **0.485 (0.012)** | *-0.053 (0.011)* | **-0.124 (0.011)** | *0.250 (0.057)* |

SNPs are those associated with Alzheimer’s disease (ALZ) risk at a Bonferroni-corrected level of significance ($p < 0.05/182 = 0.0003$). Associations with lipid fractions (low-density lipoprotein cholesterol [LDL-c], high-density lipoprotein cholesterol [HDL-c] and triglycerides) are in standard deviation units. Associations with age-related macular degeneration are log odds ratios from logistic regression analyses. Bold face indicates association at a genome-wide level of significance ($p < 5 \times 10^{-8}$); italics indicates association at a Bonferroni-corrected level of significance. All variants are orientated to the Alzheimer’s disease risk-increasing allele.

Supplementary Figure 1: Genetic associations with lipid fractions and disease outcomes

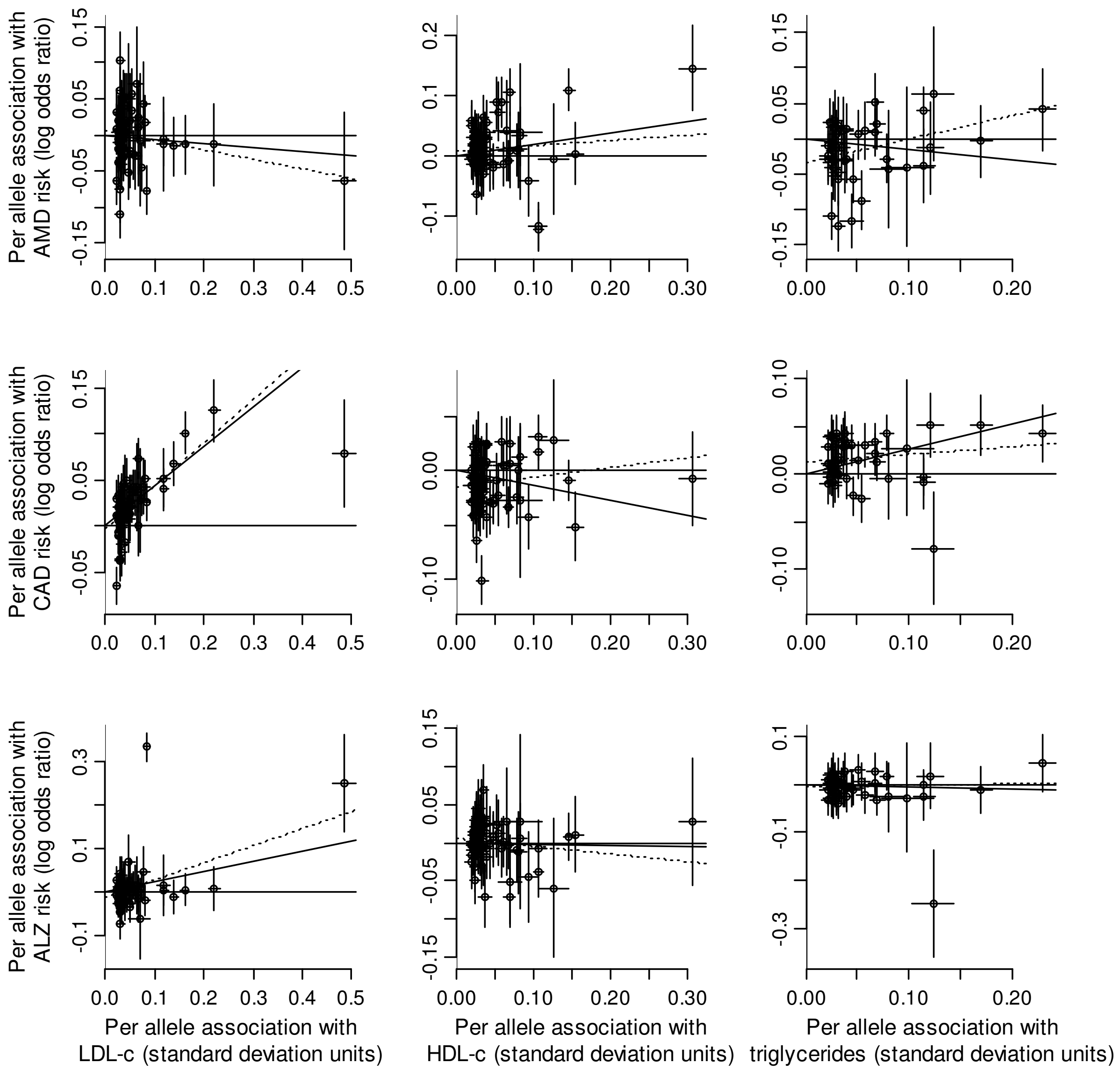


Genetic associations for all variants associated with the lipid fraction at a genome-wide level of significance ($p < 5 \times 10^{-8}$). Each point represents a single genetic variant, lines are 95% confidence intervals for the associations. The solid line is the inverse-variance weighted estimate, the dotted line is the MR-Egger estimate. This figure is also available as an interactive graph online at http://mendelianrandomization.com/amd_lipids_motionchart.htm.

Supplementary Figure 2: Distribution of causal estimates from univariable Mendelian randomization analysis of HDL-cholesterol on AMD risk on omission of 30% of the variants from the analysis at random based on 1 million iterations

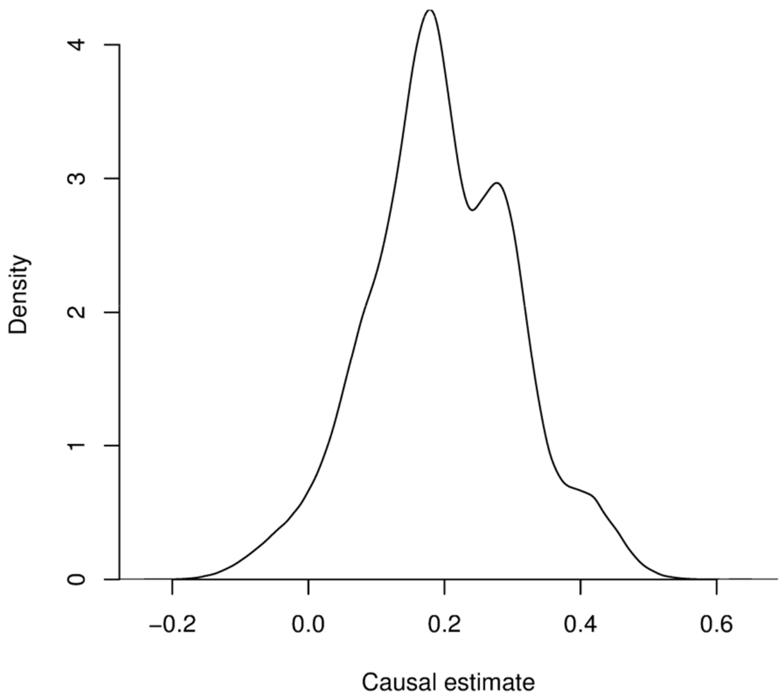

Supplement: Tables S1 and S2 and Figures S1 and S2 [file mmc1.pdf]
